# Efficacy of single high-dose statin prior to percutaneous coronary intervention in acute coronary syndrome: a systematic review and meta-analysis

**DOI:** 10.1186/s43044-024-00481-7

**Published:** 2024-04-17

**Authors:** Bryan Gervais de Liyis, Gusti Ngurah Prana Jagannatha, Anastasya Maria Kosasih, I. Kadek Susila Surya Darma, I. Made Junior Rina Artha

**Affiliations:** 1 Faculty of Medicine, Universitas Udayana, Prof. I.G.N.G Ngoerah General Hospital, Diponegoro Street, Denpasar, Bali 80114 Indonesia; 2 Department of Cardiology and Vascular Medicine, Faculty of Medicine, Universitas Udayana, Prof. I.G.N.G Ngoerah General Hospital, Denpasar, Bali Indonesia

**Keywords:** Acute coronary syndrome, Atorvastatin, Mortality, Percutaneous coronary intervention, Rosuvastatin, Single high-dose statin

## Abstract

**Background:**

The impacts of single high-dose statin preloading in patients undergoing percutaneous coronary intervention (PCI) have not been fully examined. This study aims to evaluate post-procedure impacts of single high-dose statin pretreatment with acute coronary syndrome (ACS).

**Methods:**

The meta-analysis reviewed Cochrane, PubMed, and Medline databases for studies comparing single high-dose atorvastatin or rosuvastatin to placebo in ACS patients undergoing PCI. The primary endpoints included major adverse cardiovascular events (MACE), myocardial infarction (MI), all-cause mortality, and target vessel revascularization (TVR) at three months. Secondary endpoints examined were the TIMI flow grade 3 and left ventricular ejection fraction (LVEF).

**Results:**

Comprehensive analysis was conducted on fifteen RCTs, encompassing a total of 6,207 patients (3090 vs 3117 patients). The pooled results demonstrated that a single high-dose of statin administered prior to PCI led to a significant decrease in the incidence of MACE at three months post-PCI compared to the control group (OR 0.50, 95%CI 0.35–0.71, *p* = 0.0001). The occurrence of MI (OR 0.57, 95%CI 0.42–0.77, *p* = 0.0002), all-cause mortality (OR 0.56, 95%CI 0.39–0.81, *p* = 0.0002), and TVR (OR 0.56, 95%CI 0.35–0.92, *p* = 0.02) was significantly lower in the statin single high-dose group compared to the control group. No significant effects on TIMI flow grade 3 (OR 1.20, 95%CI 0.94–1.53, *p* = 0.14) or left ventricular ejection fraction (OR 2.19, 95%CI − 0.97 to 5.34, *p* = 0.17) were observed. Subgroup analysis demonstrated reduced incidence of MACE with a single dose of 80 mg atorvastatin (OR 0.66, 95%CI 0.54–0.81, *p* < 0.0001) and 40 mg rosuvastatin (OR 0.19, 95%CI 0.07–0.54, *p* = 0.002).

**Conclusions:**

Single high-dose statin before PCI in patients with ACS significantly reduces MACE, MI, all-cause mortality, and TVR three months post-PCI.

## Background

Percutaneous coronary intervention (PCI) is a procedure with a variety of indications, spanning from acute coronary syndrome (ACS) to elective revascularization [1]. Nevertheless, major adverse cardiac events (MACE) both pre-procedure as well as post-procedure associated with PCI itself or resulting from ACS persist. Therefore, it is imperative to employ appropriate interventions to optimize the outcomes of ACS patients undergoing PCI. The effectiveness of several strategies to reduce periprocedural MACE, including ticlopidine [2], eptifibatide [3], and clopidogrel [4], have been previously investigated. Furthermore, there is mounting evidence suggesting a promising effect of pretreatment with statins in patients with chronic coronary syndrome (CCS) or ACS for this purpose [5, 6]. Interestingly, the advantageous outcomes emanate from statins, extending beyond their conventional impact on lipid levels [7–13].

At present, guidelines recommend the use of high-dose statins both before and after PCI in ACS patients [14]. However, the optimal timing for initiating statin therapy and the benefits of a single high-dose statin administration prior to PCI in ACS patients concerning MACE remain unclear. The largest randomized controlled trial (RCT) conducted to date, evaluating the effects of loading high-dose high-intensity statins before PCI in ACS patient populations, has yielded diverse outcomes. Nevertheless, an extensive reduction in MACE was predominantly seen in patients undergoing PCI, particularly patients with ST-segment elevation myocardial infarction (STEMI). Given the existing knowledge gap on this matter, the aim is to analyze the use of a single high-dose statin before PCI to reduce MACE following the procedure.

## Methods

### Research design

The study protocol secured registration and approval within the PROSPERO database (ID: CRD42023445800) before commencing the systematic search based on the guidelines outlined by PRISMA. The inclusion criteria for the meta-analysis encompassed randomized controlled trials focusing on the efficacy of single high-dose statin compared to placebo administered prior to PCI in adults diagnosed with ACS. The literature search, data extraction, and bias evaluation were performed solely by the author, with any divergences in the determination of study eligibility were systematically reconciled through a collaborative consensus-building process with another member.

The selected criteria were as follows: studies were required to clearly specify the type of statin used as the intervention, furnish direct comparisons of outcomes between single high-dose statin and placebo, administer either a single dose of 80 mg atorvastatin or a single dose of 20/40 mg of rosuvastatin, administer the statin no later than one week before the PCI procedure, placebo should not be any form of statin, include participants with a confirmed diagnosis of ACS based on clinical and laboratory assessments, and allocate participants equally (1:1) through randomization. Excluded from the analysis were studies with a follow-up duration of less than three months, studies involving pediatric populations, studies including post-chemotherapy participants, studies with participants exhibiting autoimmune or psychiatric conditions, and studies lacking specification of the type and dosage of statin utilized. Studies assessing patients with unstable angina were also excluded to maintain a more homogenous study population. Moreover, studies that utilized a lower dose of statin in the placebo group were not included.

### Literature search

**Fig. 1:**
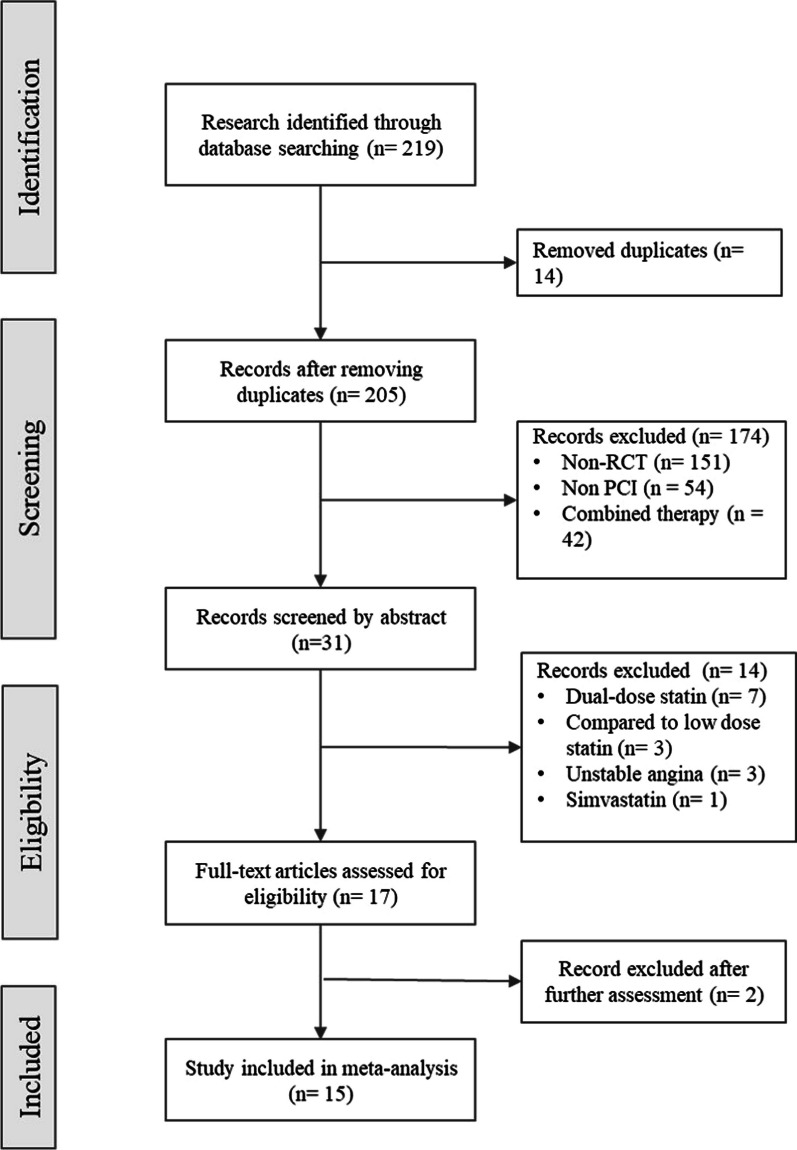
PRISMA flowchart diagram

A systematic literature search was undergone, employing Cochrane, Medline, and PubMed archives, covering the period from January 1, 2009 to January 1, 2023. Language restrictions were not applied during the search. The search strategy encompassed Medical Subject Headings (MeSH) terms and relevant free-text keywords, including ((((((((Single High-Dose Statin) OR (Statin)) OR (High-dose statin)) OR (Single dose statin)) OR (atorvastatin)) OR (rosuvastatin)) OR (high-intensity statin)) OR (high-dosage statin)) AND ((Prior) OR (Before)) AND ((((((Percutaneous coronary intervention) OR (Coronary Angioplasty)) OR (Coronary Stenting)) OR (stenting)) OR (Transluminal coronary angioplasty)) OR (Percutaneous transluminal coronary angioplasty)) AND (((((((((Acute coronary syndrome) OR (Acute myocardial infarction)) OR (Myocardial infarction)) OR (Coronary heart disease)) OR (Acute coronary event)) OR (STEMI)) OR (NSTEMI)) OR (Acute ischemic coronary syndrome)) OR (Acute coronary artery syndrome)). Two hundred and nineteen manuscripts initially identified, 17 conformed to the predetermined inclusion criteria, delineated in the PRISMA flowchart (Fig. 1). Ultimately, 15 studies were considered appropriate for integration into the quantitative analysis. Furthermore, the references of included studies were examined to unveil any additional literature pertinent to the subject.

### Quality evaluation of the included studies

A comprehensive evaluation of potential bias was conducted using the Cochrane Collaboration’s tool for Risk of Bias Assessment, comprising seven key components. Critical factors such as randomization procedures, allocation concealment, and blinding of participants were carefully evaluated to ascertain the risk of bias within the trials. The quality of evidence for all outcomes was rigorously evaluated through the implementation of the Grades of Recommendation, Assessment, Development, and Evaluation (GRADE) methodology. Integrating bias assessment with GRADE ensures a robust evaluation of evidence quality, enhancing research reliability.

### Data extraction

A rigorous and methodical data extraction process was undertaken to acquire comprehensive sociodemographic, baseline, and outcome-related information from the included studies. This process encompassed assessment of key parameters, including the geographical locations, age distribution, gender representation, statin dosage, timing of statin administration, and the specific type of ACS under investigation. The research outcomes were predicated on paramount indicators, notably major adverse cardiovascular events (MACE), myocardial infarction (MI), all-cause mortality, and target vessel revascularization (TVR) within a three-month timeframe. Additionally, other crucial parameters, such as TIMI flow grade 3 and left ventricular ejection fraction (LVEF), were employed to comprehensively assess the efficacy of statin preloading prior to PCI. These metrics served as integral benchmarks in gauging the impact of statin therapy in the context of PCI. In order to assess the specific effects of different statin types, the study conducted subgroup analyses on MACE based on the distinct types of statin utilized, namely atorvastatin and rosuvastatin. This methodological approach facilitated a refined evaluation of the efficacy of each statin type in relation to the desired clinical outcomes. By employing subgroup analysis, potential variations in treatment response between the different statins were effectively elucidated, thereby enhancing the precision and depth of the research findings.

### Data synthesis and analysis

Binary outcomes were transformed into odds ratios (ORs) along with the corresponding 95% confidence intervals (CIs). To present the results in a visually informative manner, forest plots were generated, enabling a clear and concise representation of the effect estimates and their associated CIs for each individual study. Moreover, funnel plots were constructed to assess the potential presence of publication bias, a critical consideration in meta-analyses. Heterogeneity between studies was evaluated using the I2 statistic, which quantifies the proportion of total variation attributed to between-study heterogeneity. If the I2 value exceeded 50%, indicating substantial heterogeneity, the random-effects model was employed. Conversely, if the I2 value was below 50%, suggesting low heterogeneity, the fixed-effects model was used to synthesize the data. Review Manager software version 5.4.1, a widely recognized and reliable tool for conducting meta-analyses, was employed to carry out all statistical analyses. A predetermined significance level of *p* < 0.05 was set to determine the statistical significance of the results, ensuring the attainment of rigorous and clinically meaningful findings. This statistical approach allowed for a comprehensive and nuanced exploration of the data, contributing to a robust and evidence-based synthesis of the research outcomes.

## Results

### Selection of studies

The PRISMA flow diagram in Fig. 1 shows the study selection process. The initial research yielded a total 219 studies, and through the elimination of duplications, 205 studies underwent independent screening. One hundred and eighty-eight studies were excluded due to following reason: non-randomized controlled trials, non-PCI studies, combined treatment intervention (between atorvastatin and rosuvastatin), used dual-dose statin, compared with low dose statin, patients who suffered UAP, studies that used simvastatin as intervention. After exclusion, 17 full-text studies were assessed for the eligibility. At the end, 15 studies [15–29] were included in our data synthesis.

### Characteristics of included studies and participants

Characteristics of included studies are presented in Table 1. The majority of studies were conducted in Asia [17–24, 28, 29]. Nine studies [16, 17, 19, 21, 23–27] used atorvastatin medication before PCI, while five studies [18, 20, 22, 28, 29] used rosuvastatin. One study [15] used either atorvastatin or rosuvastatin. The control groups, in their entirety, did not receive a high single dose of statin as a pretreatment prior to the PCI procedure. The intervention group consisted of 3090 patients, while the control group comprised 3117 patients. In all studies utilizing atorvastatin, a single 80 mg dose was administered; however, two studies [18, 28] opted for a 20 mg single dose, and four studies [15, 20, 22, 29] favored a 40 mg single dose of rosuvastatin. The mean age in the population receiving statins was 59.78 ± 3.4 years. Additionally, the average duration of follow-up in studies that reported this information was approximately 6.28 months. A majority of the included studies predominantly featured patients with STEMI [15, 17, 19–23, 26, 27], with a single study [28] incorporating patients with NSTEMI. The remaining studies [16, 18, 24, 25, 29] encompassed a broader spectrum of ACS patients without specific categorization by ACS subtype. Only five studies reported side effects of statins [18–20, 22, 26] with serious side effects reported in only 0.96% of these studies.

**Table 1:** Characteristics of selected studies

Studies	Countries	Clinical feature	Type of statin	Dosage	Patients (*n*)	Placebo (*n*)	Mean Age (years)	Mean, SD baseline LDL levels in intervention group (mg/dl)	Mean, SD baseline LDL levels in control group (mg/dl)	Timing of statin therapy before PCI (days)	Timing descriptions	Side effects (n, descriptions)	Follow up (month)
Adel et al. [15]	Egypt	STEMI	Atorvastatin or Rosuvastatin	Single dose 80 mg (Atorvastatin) or 40 mg (Rosuvastatin)	66	33	53.2	NA	NA	0	at ER prior primary PCI	NA	12
Briguori et al. [16]	Italy	ACS	Atorvastatin	Single dose 80 mg	338	330	55.4	126, 35	129, 37	0	at ER prior primary PCI	NA	6
Chen et al. [17]	China	STEMI	Atorvastatin	Single dose 80 mg	76	80	64	105.18, 17.78	105.57, 28.61	1	The day before the PCI	NA	NA
Guo et al. [18]	China	ACS	Rosuvastatin	Single dose 20 mg	47	45	60.71	NA	NA	0	1.5 h prior PCI	NA	12
Hahn et al. [19]	Korea	STEMI	Atorvastatin	Single dose 80 mg	89	84	57.8	NA	NA	0	after PCI	0, adverse drug reactions or liver functional damage	6
Kim et al. [20]	Korea	STEMI	Rosuvastatin	Single dose 40 mg	213	267	55.5	117.7, 34.4	118.7, 36.8	0	within 12 h after symptom onset	10. ALT > 3 times the upper normal limit (but prevalence in control group is 7.1% with p-value 0.49)	1
Kim et al. [21]	Korea	STEMI	Atorvastatin	Single dose 80 mg	30	37	62.2	NA	NA	0	before primary PCI	0, No serious side effects were detected associated with rosuvastatin loading	6
Ko et al. [22]	Korea	STEMI	Rosuvastatin	Single dose 40 mg	92	93	57.4	NA	NA	0	as early as possible after randomization	NA	3
Liu et al. [23]	China	STEMI	Atorvastatin	Single dose 80 mg	32	32	57.7	NA	NA	0	emergency room before primary PCI	0, All patients tolerated the study drugs well without side effects	12
Liu et al. [24]	China	ACS	Atorvastatin	Single dose 80 mg	400	398	59.3	150.8, 34.8	150.8, 42.54	0	before emergency PCI	NA	12
Lopes et al. [25]	Brazil	ACS	Atorvastatin	Single dose 80 mg	1351	1359	61.8	NA	NA	0	12 h before elective PCI or with other antiplatelet drugs before urgent/emergent PCI	NA	1
Mendez et al. [26]	Brazil	STEMI	Atorvastatin	Single dose 80 mg	49	54	64	NA	NA	0	prior to primary PCI	NA	1
Post et al. [27]	Netherlands	STEMI	Atorvastatin	Single dose 80 mg	20	22	61.7	130.7, 30.9	110.98, 21.6	0	The timing of study medication administration varied according to type of ACS. For patients with ACS without ST-segment elevation, the first dose was administered between 2 and 12 h before angiography and PCI. For patients with ST-segment elevation MI (STEMI), the first loading dose was administered as soon as possible before primary PCI	0, No cases of rhabdomyolysis or hepatic failure were reported in the atorvastatin group. Creatine phosphokinase and aminotransferases levels were not significantly different in patients treated with atorvastatin vs placebo	3
Wang et al. [28]	China	NSTE-ACS	Rosuvastatin	Single dose 20 mg	62	63	57.5	96.67, 34.8	92.8, 34.8	0	before primary PCI	NA	1
Yun et al. [29]	Korea	ACS	Rosuvastatin	Single dose 40 mg	225	220	65.4	122, 38	124, 40	NA	before PCI	NA	12

*ACS* acute coronary syndrome, *NSTE-ACS* non ST-elevation acute coronary syndrome, *STEMI* ST-elevation myocardial infarction

### Quality evaluation of the included studies

**Fig. 2:**
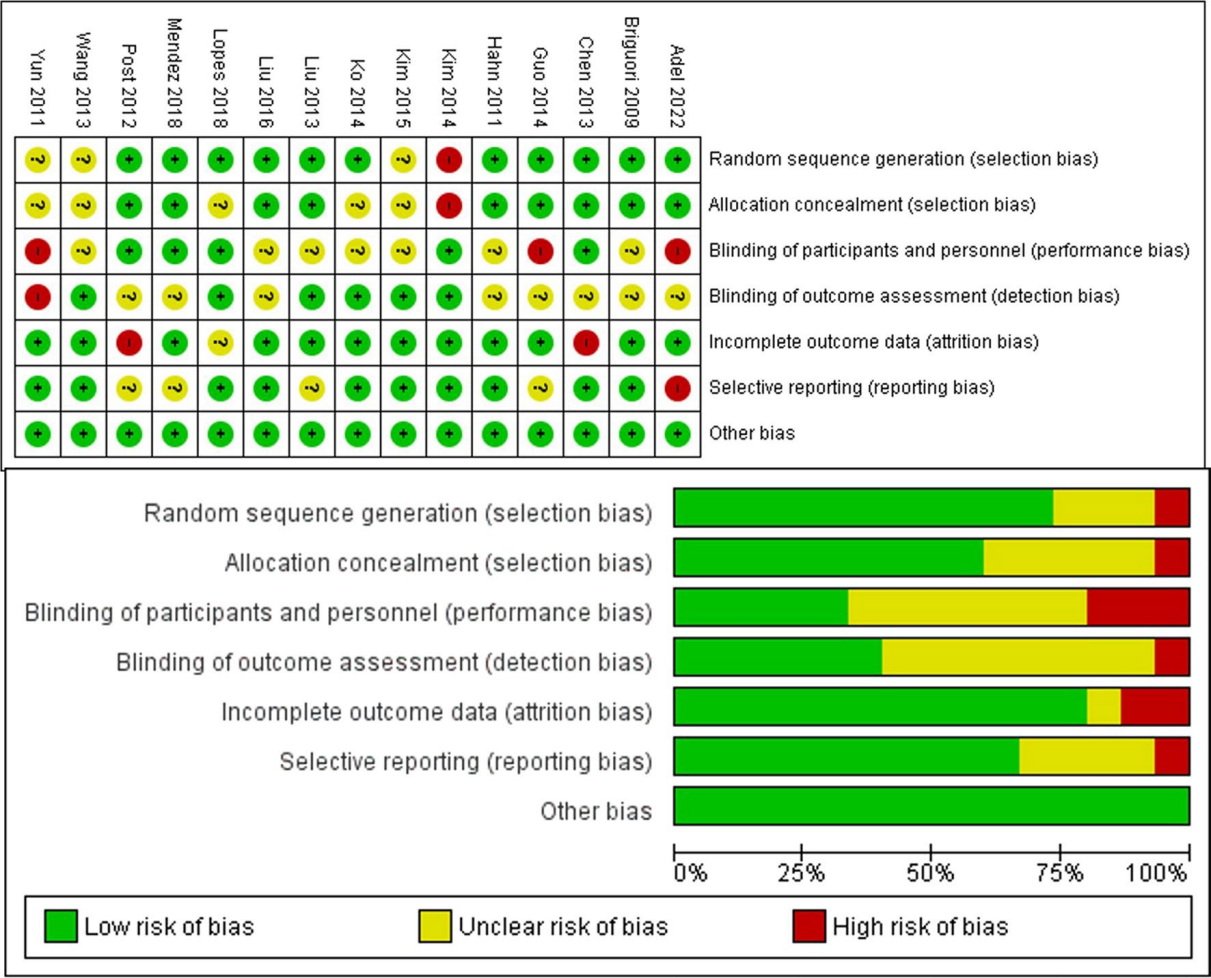
Risk of bias summary and graph

**Fig. 3:**
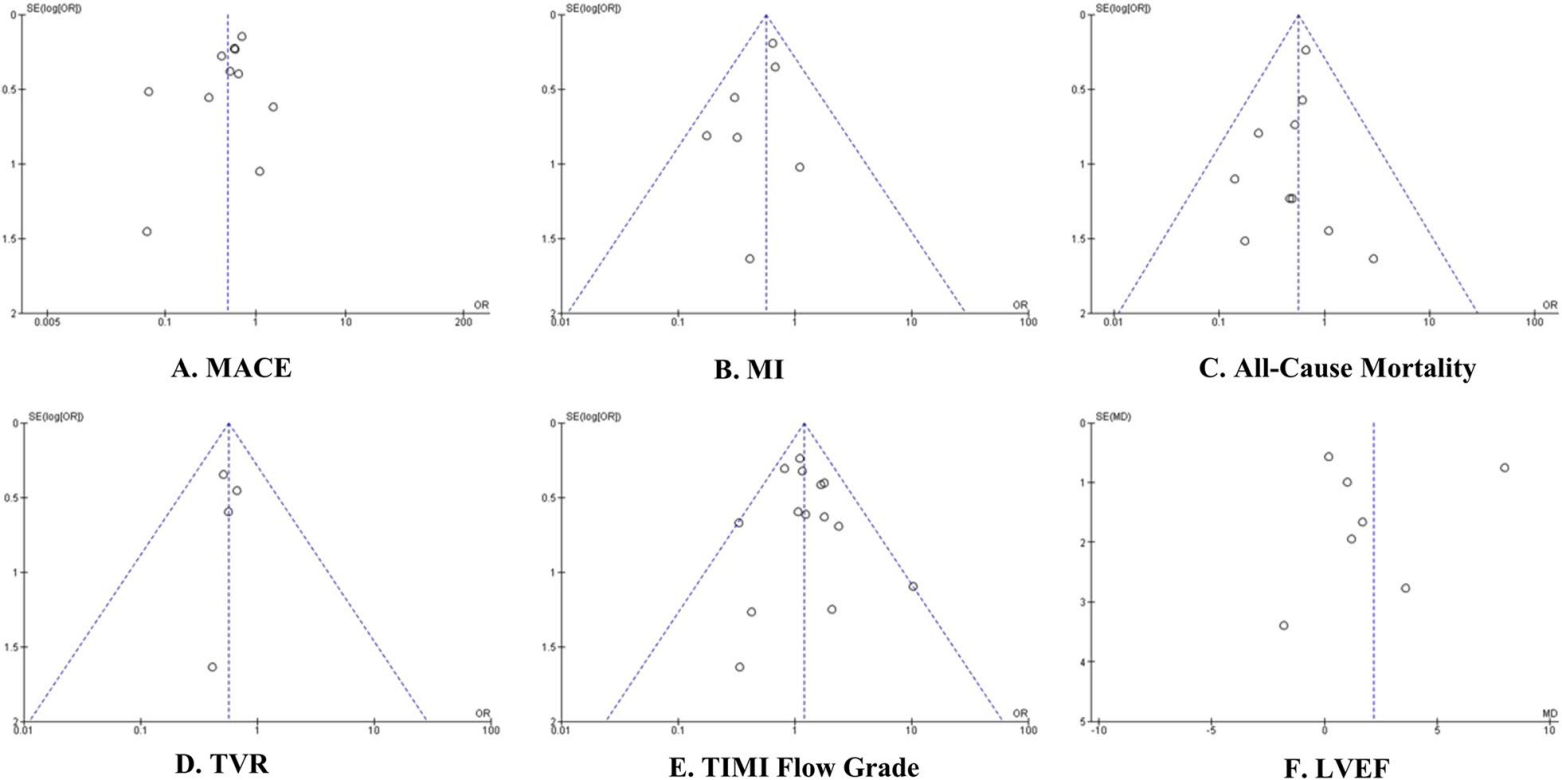
Funnel plots of included studies in terms of **A** Major adverse cardiovascular events (MACE), **B** Myocardial infraction (MI), **C** All-cause mortality, **D** Target vessel revascularization (TVR), **E** TIMI Flow Grade 3, and **F** Left ventricular ejection fraction (LVEF). Abbreviations: MACE; major adverse cardiovascular events, MI; myocardial infraction, TVR; target vessel revascularization, LVEF; left ventricular ejection fraction

The collective quality of the encompassed studies, as assessed through the Cochrane risk of bias evaluation, fell within the classification of low bias quality (Fig. 2). Six studies [15, 17, 18, 20, 27, 29] were categorized as having high risk of performance bias. However, it is worth noting that all the studies were categorized as having an unclear bias, particularly in the domain of detection bias, attributed to unexplained factors influencing the outcome assessment. For each outcome, we employed funnel plots to detect bias. As illustrated in Fig. 3, the funnel plots for all-cause mortality, MI, and TVR outcomes exhibited a symmetrical pattern, signifying a very low risk of bias (all *I*2 = 0%). Another outcome with a low risk of bias was observed in the TIMI Flow Grade (*I*2 = 5%). In contrast, funnel plots for the MACE and LVEF outcomes displayed asymmetry, indicating heterogeneous results among the included studies (*I*2 = 63% and *I*2 = 92%, respectively).

### Efficacy of single high-dose statin prior to PCI

**Fig. 4:**
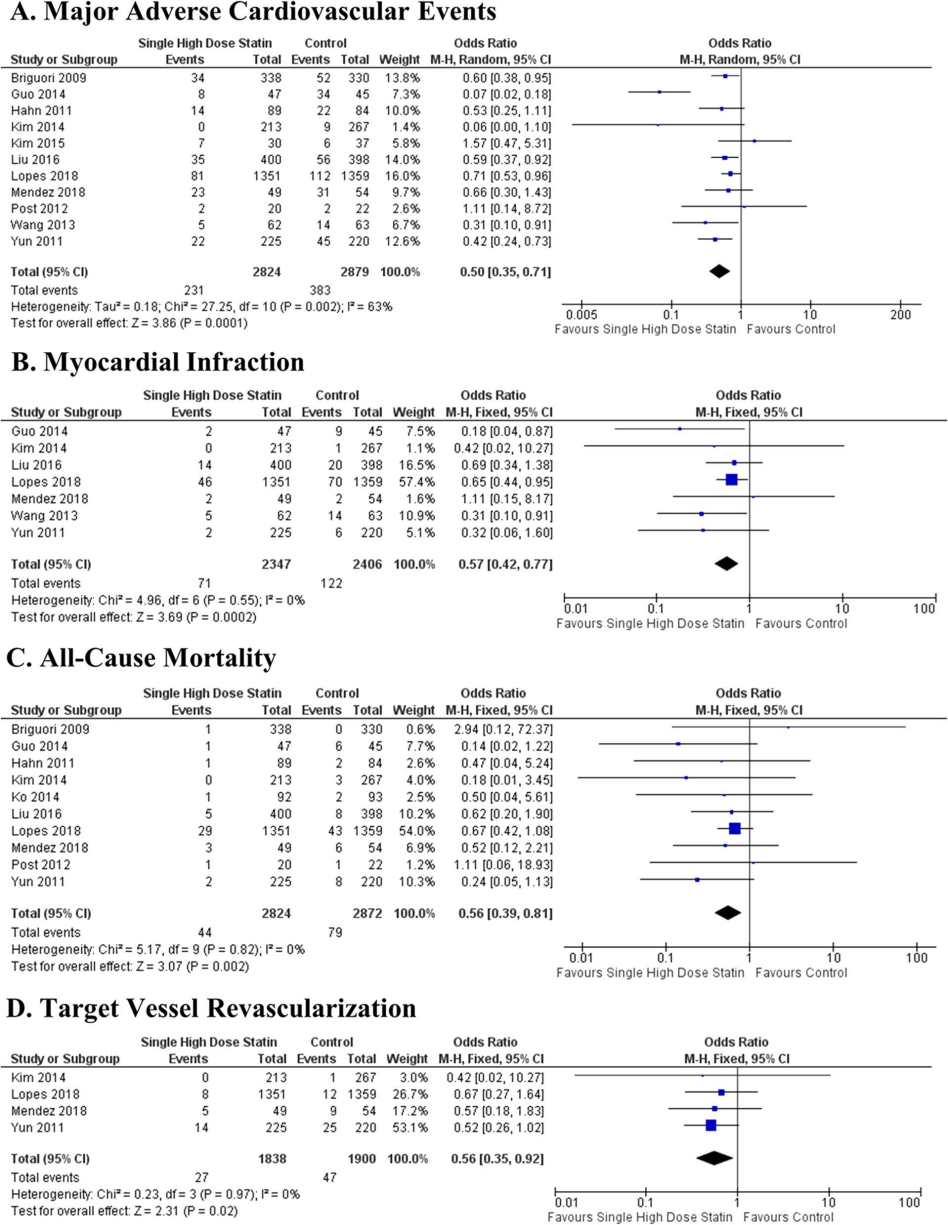
Effects of single high-dose statin pre-PCI on **A** Major adverse cardiovascular events (MACE), **B** Myocardial infarction (MI), **C** All-cause mortality, and **D** Target vessel revascularization (TVR) post-PCI compared to placebo

**Fig. 5:**
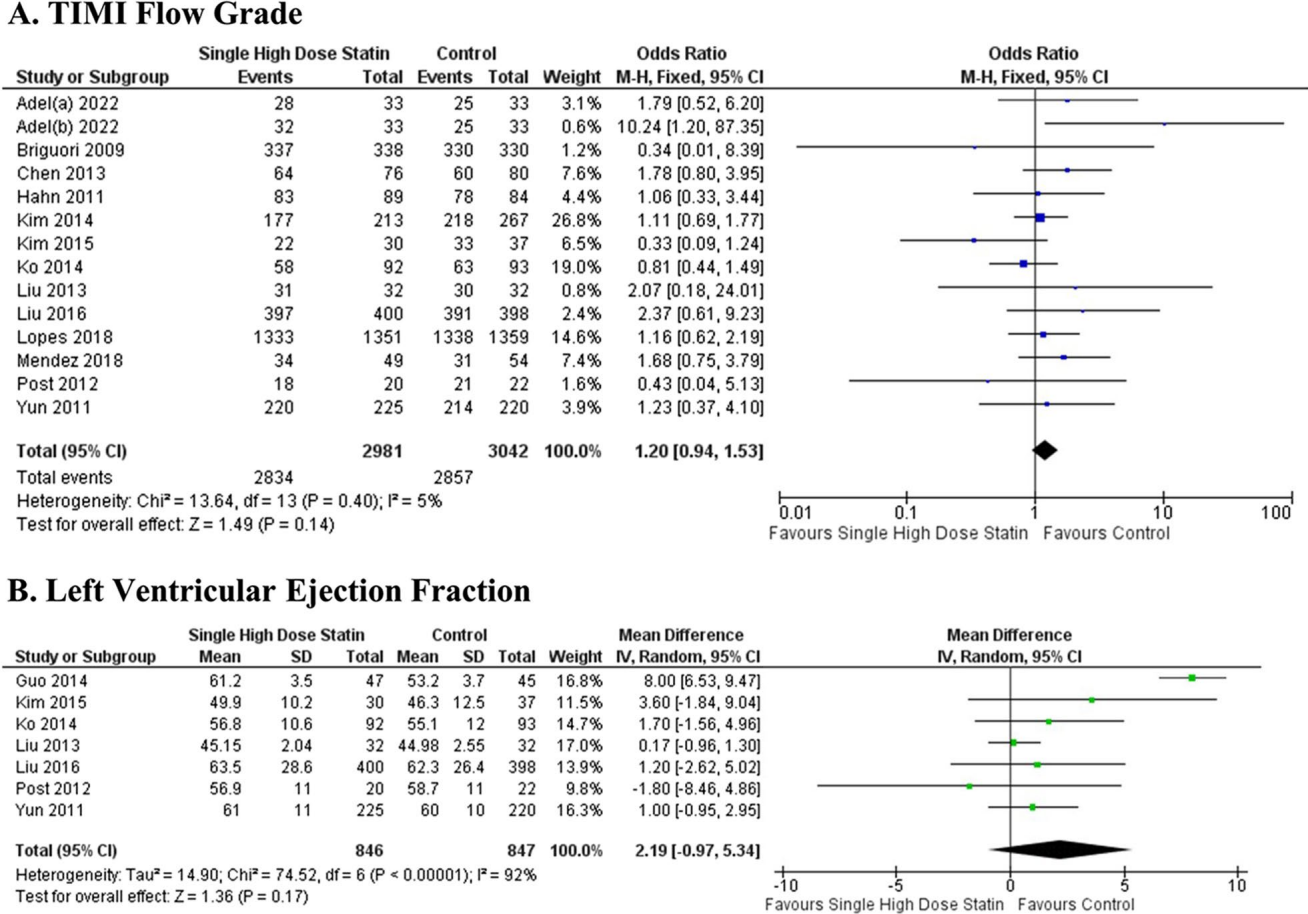
Effects of single high-dose statin pre-PCI on **A** TIMI flow grade 3 and **B** Left ventricular ejection fraction (LVEF) post-PCI compared to placebo

The results of the analysis demonstrated that the administration of a single high-dose statin prior to PCI procedure significantly reduced the occurrence of MACE when compared to the control group (OR 0.50; 95% CI [0.35–0.71]; *P* < 0.001; *I*2 = 63%; Fig. 4A). Furthermore, the single high-dose statin group exhibited fewer instances of MI following the PCI procedure and a lower rate of all-cause mortality (OR 0.57; 95% CI [0.42–0.77]; *P* < 0.001; *I*2 = 0% and OR 0.56; 95% CI [0.35–0.92]; *P* < 0.001; *I*2 = 0%, respectively; Fig. 4B, C). The high-dose statin group also displayed a significant decrease in TVR post-PCI within a 3-month timeframe when compared to the control group (OR 0.56; 95% CI [0.35–0.92]; *P* = 0.02; *I*2 = 0%; Fig. 4D). However, the efficacy of statin preloading before PCI, as assessed by TIMI flow grade 3 and LVEF, showed no significant differences (*P* = 0.14 and *P* = 0.17; respectively; Fig. 5A, B).

**Fig. 6:**
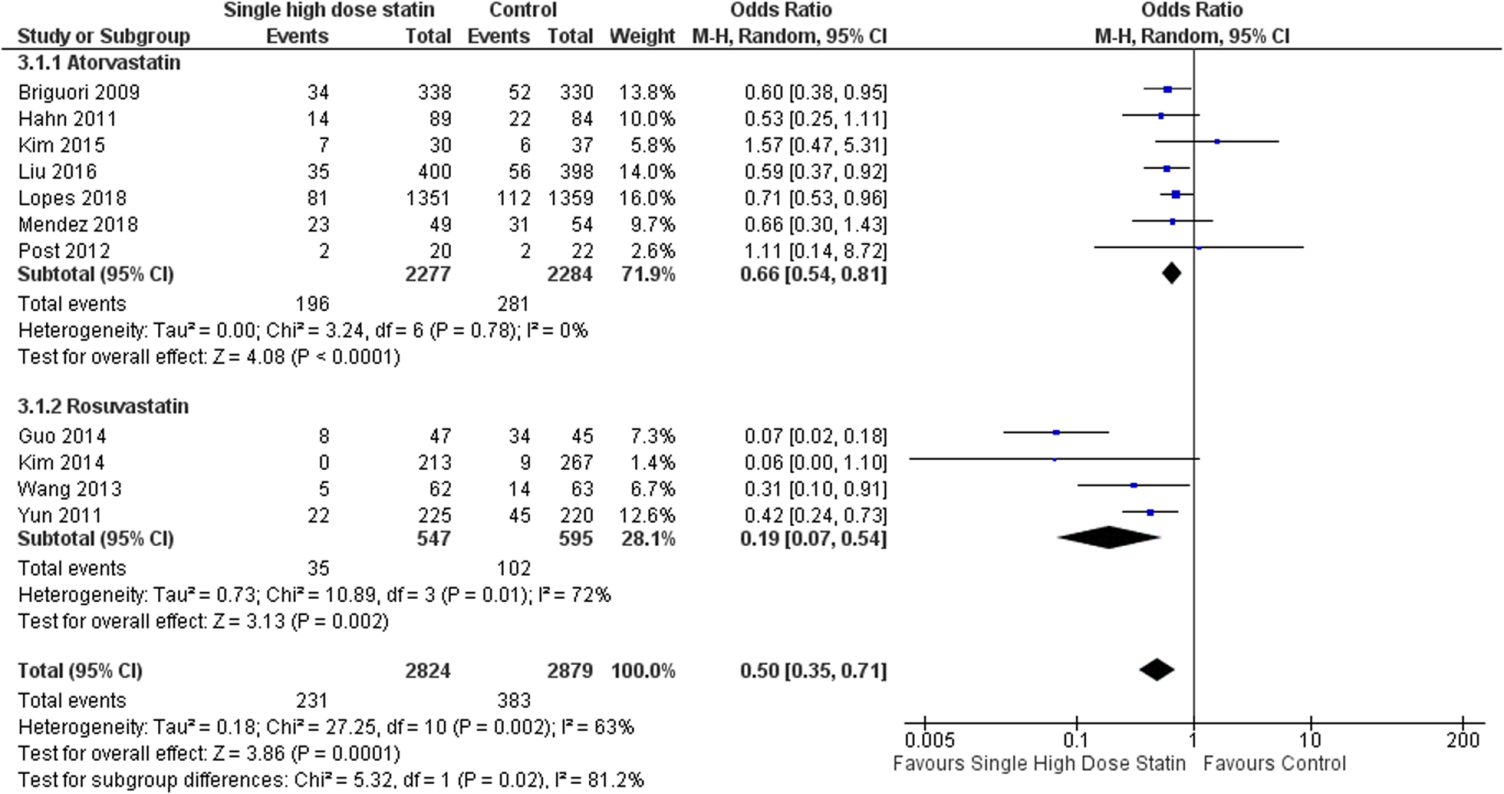
Subgroup analysis of single high-dose statin pre-PCI in MACE based on the type of statin compared to placebo

**Fig. 7:**
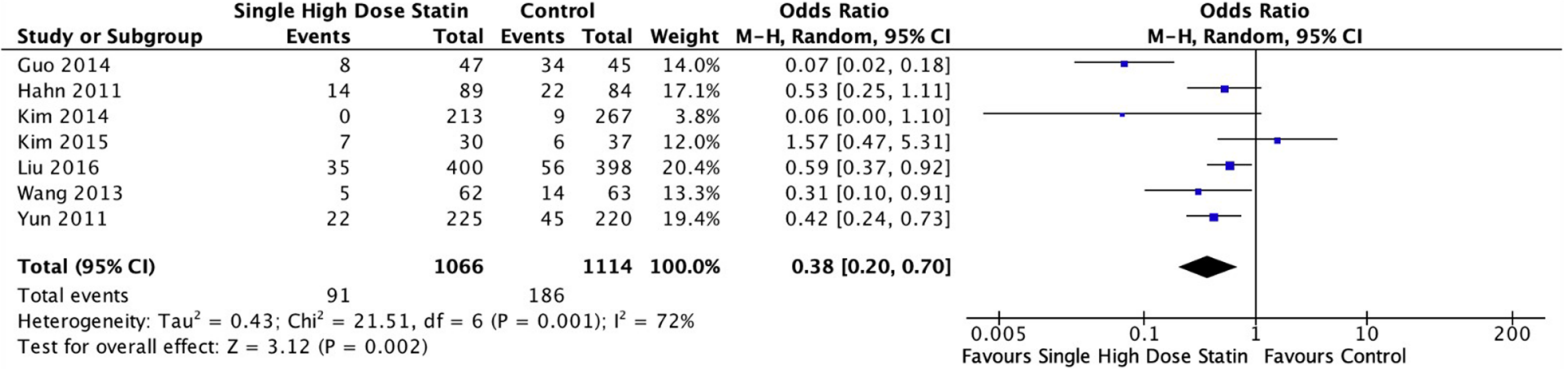
Subgroup analysis of single high-dose statin pre-PCI in MACE based on Asian population

The MACE outcome for each statin group and based on Asian population were further evaluated through subgroup analysis. Patients receiving Atorvastatin 80 mg displayed a notable 0.6 times reduction in the risk of MACE within 3 months of PCI (OR 0.66; 95% CI [0.54–0.81]; *P* < 0.001; *I*2 = 0%; Fig. 6). Similarly, the administration of Rosuvastatin 40 mg also significantly reduced the risk of MACE by 0.19 times after PCI (OR 0.19; 95% CI [0.07–0.54]; *P* = 0.002; *I*2 = 72%; Fig. 6). In the Asian population, single high-dose statin before PCI consistently reduced the risk of MACE (OR 0.38; 95% CI [0.20–0.70]; *P* = 0.002; *I*2 = 72%; Fig. 7). A summary of the forest plot detailing the effects of a single high-dose statin prior to PCI compared to the control group is presented in Table 2.

**Table 2:** Forest plots summary

Endpoints	Single high-dose statin	Placebo	Odds ratio/ mean differences [95% CI]	*P* values
Major adverse cardiovascular events (MACE)	8.18% (231/2824)	13.30% (383/2879)	OR 0.50 [0.35–0.71]	0.0001*
Myocardial infraction (MI)	3.03% (71/2347)	5.07% (122/2406)	OR 0.57 [0.42–0.77]	0.0002*
All-cause mortality	1.56% (44/2824)	2.75% (79/2872)	OR 0.56 [0.39–0.81]	0.0002*
Target vessel revascularization (TVR)	1.47% (27/1838)	2.47% (47/1900)	OR 0.56 [0.35–0.92]	0.02*
TIMI flow grade 3	95.07% (2834/2981)	93.92% (2857/3042)	OR 1.20 [0.94–1.53]	0.14
Left ventricular ejection fraction (LVEF)	–	–	OR: 2.19 [-0.97–5.34]	0.17
MACE subgroup analysis				
80 mg atorvastatin	8.61% (196/2277)	12.30% (281/2284)	OR 0.66 [0.54–0.81]	< 0.0001*
40 mg rosuvastatin	6.40% (35/547)	17.14% (102/595)	OR 0.19 [0.07–0.54]	0.002*
Asian population	8.53% (91/1066)	16.69% (186/1114%)	OR 0.38 [0.20–0.70]	0.002*

*Significant < 0.05

## Discussion

The primary findings of this meta-analysis, encompassing 6207 patients from 15 RCTs, reveal that single high-dose statin administration before PCI significantly decreases MACE after the procedure in the ACS population. This benefit is consistent for both Atorvastatin 80 mg and Rosuvastatin 40 mg, which are high-intensity statins. Compared to our meta-analysis, previous meta-analyses, although being more heterogeneous, conducted by Patti et al. [30], Wang et al. [5], Benjo et al. [31], dan Soud et al. [32] have shown that high-intensity statin pretreatment can substantially reduce MACE in patients undergoing PCI. This conclusion aligns with our meta-analysis, indicating lower incidence of MACE, including myocardial infarction and TVR, in cases of single high-dose statin administration before PCI. Additionally, Navarese et al. [33] showed that the effect of statin varies with the timing of administration; the earlier statins are given before PCI, the greater the benefit, and statin treatment before PCI significantly reduces the onset of myocardial infarction compared to post-PCI treatment. Soud et al. [32] emphasized that while pre-intervention statin use reduces MACE, the statistical significance of statin therapy before treatment in long-term mortality is not substantial. Conversely, our study indicates that single high-dose statin administration before PCI also provides benefits in terms of reducing all-cause mortality. This is likely due to all-cause mortality in our study being predominantly influenced by cardiovascular death, given that our study population specifically comprises ACS patients, who have a high 30-day mortality rate due to reinfarction compared to CCS patients.

However, loading a single high-dose statin prior to PCI was not significantly associated with achieving TIMI flow grade 3 or LVEF values compared to the control. Previous meta-analyses have demonstrated the benefits of loading a single high-dose statin before PCI in preventing the no-reflow phenomenon in the ACS population [34]. However, this cannot be equated with the attainment of TIMI flow grade 3, as the primary goal of PCI itself is to achieve TIMI flow grade 3 [1]. Hence, it is apparent that the administration of any medication would likely have minimal impact, as the primary goal of PCI is inherently to attain TIMI flow grade 3. This can be observed in our analysis where the proportion of achieving TIMI flow grade 3 in both groups was equally high (95% vs. 94%). The lack of a significant improvement in LVEF, in contrast to the reduction in MACE in this study, is not surprising, considering that the follow-up times of the included studies were relatively short and dominated by preserved baseline LVEF, which may mask the benefits of statins. Consistent with this, the benefits of a single high-dose statin prior to PCI in improving LVEF were observed in studies with lower baseline LVEF and longer follow-up periods [18, 23]. Conversely, Adel et al. [35] reported a higher LVEF in the single high-dose statin prior to PCI group at a shorter observation period (at discharge). However, it should be noted that this study did not report baseline LVEF in both groups, which could potentially introduce bias in interpreting these results, and thus, it was not included in the LVEF analysis.

The observed independent benefit of reducing MACE by single high-dose statin prior to PCI, apart from achieving TIMI flow grade 3 and enhancing LVEF, suggests that statins contribute not merely at the level of straightforward reperfusion but at a more intricate biomolecular level, as previously proposed by several studies [13, 36, 37]. The full extent of cardioprotective profiles from early, high-dose statin administration in ACS patients undergoing PCI remains unclear. However, it is theorized that statins offer positive pleiotropic effects beyond lipid-lowering [7]. The CANTOS trial revealed that blocking the interleukin-1β inflammatory pathway with monoclonal antibodies reduced recurrent cardiovascular events in individuals with prior history of myocardial infarction and heightened systemic inflammation, showed by the values of high-sensitivity C-reactive protein (CRP) [38]. Medications that interfere with inflammation and immunity pathways, such as colchicine, methotrexate, and IL-6 receptor antagonists, have been investigated for MACE prevention with varying degrees of success in clinical trials [39]. Statins exhibit anti-inflammatory properties and lower CRP levels independently of reducing low-density lipoprotein (LDL) [40]. The combined anti-inflammatory and lipid-lowering actions of early high-dose statin administration may provide protection against MACE, even though these mechanisms are not yet fully explained. This is supported by the included studies that also assessed changes in various biomarkers, reporting a linear decrease in MACE in the single high-dose statin prior to PCI group alongside reductions in inflammatory and remodeling biomarkers such as CRP, high-sensitivity CRP, pro-brain natriuretic peptide, cardiac troponin I, CK-MB, and matrix metalloproteinase-9 [16, 18, 23].

Regrettably, despite the significant reduction in MACE with single high-dose statin prior to PCI, there is evidence of differing responses to this treatment among ACS subtypes. In the study by Lopes et al. [25], high-dose atorvastatin significantly reduced MACE by up to 34% at 30 days (HR 0.66, 95% CI 0.48–0.98), but this benefit was observed primarily in the STEMI subtype and not in NSTE-ACS patients. Similarly, the greater benefit of rosuvastatin compared to atorvastatin in reducing MACE in the NSTE-ACS population may be attributed by rosuvastatin’s lower incidence of global and capillary inflammatory activities in ACS patients [41]. This improvement could translate into better clinical outcomes. Elevated hs-CRP values have been suggested to be a predictive marker for new MACE and cardiovascular death, as well as all-cause mortality in ACS patients [42]. Still, little is known about the molecular mechanisms behind the benefits of rosuvastatin in NSTE-ACS.

Furthermore, individuals who have previously received statin medication as well as those that are naïve to statins exhibit diversity in their differential response to the advantages of statin therapy. Wang et al. [5] and Pan et al. [43] found that high-intensity statin therapy in statin-naive patients has a protective effect on acute myocardial infarction events and tricuspid valve stenosis, while no effect was observed in patients with prior statin treatment. In contrast, Chitose et al. [44] concluded favorable effects on periprocedural myocardial infarction in patients not using statins and in individuals on long-term statin therapy. Currently, there is limited literature that can compare the outcomes of single high-dose statin prior to PCI in patients on long-term statin therapy with those not using statins. Owing to the inconsistent outcomes across different trials, further research is crucial to distinguish the effects of statins in short-term vs long-term treatment. The impact of statin usage on outcomes for PCI patients is being studied in ongoing clinical studies (NCT04974814, NCT04754789).

### Limitations

There are a number of limitations to take into account when interpreting our results. Given that the majority of the included studies were carried out in East Asia, a number of variables, including genetic variability, socioeconomic position, and regional differences, may have impacted our findings. Furthermore, we did not perform subgroup analyses based on the duration of statin use or ACS subtypes, which may yield different responses, as explained earlier. Furthermore, our study’s control cohort, which included participants receiving either a placebo, a moderate-intensity statin, or a low-intensity statin, was poorly characterized. When it comes to endpoints, we found that there was some variation in the impacts of bigger vs smaller studies, but we were able to address this by using a random effects analytical approach, which yielded findings that are more comparable and broadly applicable than those obtained using a fixed model. Concerning side effects, while serious adverse events are reported to be less than 1%, the studies documenting these side effects are limited. Therefore, the safety outcomes cannot be conclusively confirmed, particularly in the Asian population. Additionally, the presence of publication bias may be indicated by partially asymmetric funnel plots.

## Conclusions

In conclusion, our study involving patients undergoing PCI for ACS revealed that a single high-dose statin administered prior to the procedure significantly reduced the incidence of MACE, MI, all-cause mortality, and TVR at three months post-PCI when compared to the control group. This suggests that single high-dose statin preloading may offer substantial benefits in the context of ACS patients undergoing PCI. Notably, subgroup analyses further demonstrated the efficacy of 80 mg atorvastatin and 40 mg rosuvastatin in reducing the incidence of MACE. However, no significant effects were observed on TIMI flow grade 3 or left ventricular ejection fraction. These findings support the consideration of single high-dose statin preloading as a potential therapeutic strategy in ACS patients undergoing PCI.

## Data Availability

Data available within the article. The authors confirm that the data supporting the findings of this study are available within the article.

## Abbreviations

ACS: Acute coronary syndrome
CCS: Chronic coronary syndrome
CRP: C-reactive protein
GRADE: Grades of recommendation, assessment, development, and evaluation
LVEF: Left ventricular ejection fraction
MACE: Major adverse cardiac events
MI: Myocardial infarction
PCI: Percutaneous coronary intervention
RCT: Randomized controlled trial
STEMI: ST-segment elevation myocardial infarction
TIMI: Thrombolysis in myocardial infarction
TVR: Target vessel revascularization
